# Framing Political Messages to Fit the Audience’s Regulatory Orientation: How to Improve the Efficacy of the Same Message Content

**DOI:** 10.1371/journal.pone.0077040

**Published:** 2013-10-09

**Authors:** Lucia Mannetti, Ambra Brizi, Mauro Giacomantonio, E. Tory Higgins

**Affiliations:** 1 Department of Social and Developmental Psychology, University of Rome “Sapienza”, Rome, Italy; 2 Department of Psychology, Columbia University, New York, New York, United States of America; University G. d'Annunzio, Italy

## Abstract

This research investigates how the impact of persuasive messages in the political domain can be improved when fit is created by *subliminally priming* recipients’ regulatory focus (either promotion or prevention) and by linguistic framing of the message (either strategic approach framing or strategic avoidance framing). Results of two studies show that regulatory fit: a) increases the impact of a political message favoring nuclear energy on implicit attitudes of the target audience (Study 1); and b) induces a more positive evaluation of, and intentions to vote for, the political candidate who is delivering a message concerning immigration policies (Study 2).

## Introduction

The topic of voter persuasion has historically attracted academic attention in several disciplines such as journalism [1], political science [2], economics (specifically, public choices [3]), and, recently, psychology [4-6]. In recent political communication research literature, framing effects have received increased interest by showing their ability to influence attitudes and opinions among citizens and to affect how people think about issues [7-9].

Even a brief look at this literature shows that the label “frame” is used to indicate different processes. Social psychological interest has emphasized the influence of communication frames on individuals' attitudes and behaviors, consistent with the use of framing by Tversky and Kahneman [10] in the context of Prospect theory. In this tradition, a basic distinction between a "loss frame" and a "gain frame" is proposed with reference to potential outcomes of a given choice. This distinction fits well for several issues relevant to political choices, and, indeed, is often used by political sources of communication (political parties, and candidates).

The most recent work on political communication points out that the effectiveness of framing should not be conceived as unconditional and absolute but, on the contrary, is constrained and moderated by several factors such as the target's chronically activated frames [11] and the perceived credibility of the source [12]. The psychological literature also suggests another possible moderating factor for framing effects; namely, the extent to which a frame fits the target's prevailing regulatory focus. According to the Regulatory Focus theory [13,14], people regulate their own behavior by focusing their attention either on a better end-state to be attained (promotion focus) or on a satisfactory current state to be maintained (prevention focus). The dominance of one or the other of the two foci can be a function both of stable inter-individual differences and of situational factors.

It is known from persuasion and advertising research that persuasive messages are particularly effective if the content of a message suits the recipients’ regulatory focus [15-16-17-18- 19-20]. This means that whether a piece of information is perceived as more or less convincing can depend on the motivational orientation it addresses. The aim of the present studies is to assess whether political candidates’ messages, concerning different issues, can be made more effective by framing them in order to fit recipients’ regulatory orientation.

### Promotion focus vs. prevention focus and regulatory fit

Regulatory Focus theory proposes that people can pursue two distinct goals during self- regulation: promotion goals and prevention goals [13,14]. Individuals with a promotion goal orientation are concerned with attaining better states and are concerned with advancement and growth. Individuals with a prevention goal orientation, instead, are concerned with maintaining a satisfactory current state and with security and safety. Consequently, promotion-focused individuals are sensitive to the presence or absence of positive outcomes (or gains vs. non-gains) and prevention-focused individuals are sensitive to the presence or absence of negative outcomes (or losses vs. non-losses). Whereas striving for ideals (i.e., hopes, wishes and aspirations) by using eager strategies underlies goal achievement for a promotion focus, fulfilling ought (i.e., duties, obligations and responsibilities) by using vigilant strategies underlies goal achievement for a prevention focus [13-14-21]. Regulatory focus can vary across both individuals and situations. Whereas chronic regulatory focus can be established through socialization [13] or culture [22], situational regulatory focus can be induced for example by activating a person’s ideals or ought [19-23- 24].

Regulatory fit theory posits that motivational strength is enhanced when the manner in which people pursue a goal sustains their regulatory orientation [25,26]. The same desired end-state, e.g., tax compliance, can be described as being congruent with either a promotion or a prevention goal, i.e., either by emphasizing attaining a better state: “People should pay taxes in order to obtain better functioning public services”, or by emphasizing maintaining a current satisfactory state rather than a worse state: “People should pay taxes in order to avoid malfunctioning public services” [13]. Several studies have provided evidence that commercial as well as non-commercial advertisements are particularly effective under regulatory fit, i.e., if the content of a persuasive message corresponds to the recipients’ goal orientation [15-16-17-18-19- 20- 27].

Florack and Scarabis, for example, found that consumers preferred a product more strongly when the claim used in an advertisement for the product was compatible with their regulatory focus [17]. Cesario et al. showed that people more strongly approved a new education policy when the framing of a message in favor of the policy corresponded to their regulatory focus [15] (for a similar finding, [28]). Evidence for a positive influence of regulatory fit in information campaigns concerning tax issues was provided in countries with different “tax morale” such as Austria [29] and Italy [30]. Results of both these studies, in fact, show that persuasive campaigns aimed at increasing tax compliance can be more effective if they are constructed in order to create an experience of regulatory fit between the message and recipients’ regulatory focus. Finally, Boldero and Higgins explored the impact of regulatory focus in the political domain of economic decision-making, asking participants to choose between a “risky” option (changing the status quo) and a “conservative” option (maintaining the status quo) [31]. Findings of their studies show that a prevention focus was associated with strategic vigilance which in turn predicted conservative choices, whereas a promotion focus was associated with strategic eagerness which in turn predicted risk taking.

 Before presenting our research addressing political persuasion, it is worth clarifying that our distinction between *strategic* approach eagerness that fits promotion and *strategic* avoidance vigilance that fits prevention differs from – is orthogonal to – the distinction between approach activation versus avoidance inhibition which, according to work by Janoff-Bulman and her associates [32], differentiates liberal from conservative. Indeed, both strategic eagerness and vigilance can tactically use either approach activation or avoidance inhibition [33].

## Study 1

In Study 1, we wanted to test whether regulatory fit can improve the effectiveness of messages delivered by an anonymous political candidate pointing out the positive economic consequences of nuclear power production. We chose this specific topic because we intended to test the impact of regulatory fit on a very difficult-to-change attitude.

Indeed, several studies across the world have shown that there exists a strong “public resistance” toward this type of energy. Whitfield et al., after examining values, beliefs, and trust as factors underling attitude toward nuclear power, conclude that “it is relatively easy to increase nuclear power opposition with negative events such as public protests or accidents…but very difficult to increase nuclear support, even after a long period of safe operations (p. 436)” [34]. Public resistance is particularly strong in Italy where in 1987, after a referendum, nuclear power plants were deactivated. In the period of our data collection (winter 2011), a strong national debate was going on in preparation for a second referendum scheduled for June 2011.

Instead of either measuring chronic regulatory foci or manipulating them by asking participants to think about their present and past ideals or obligations, as usual in previous literature, we chose to subliminally prime promotion or prevention focus of message recipients. To our knowledge, regulatory foci have never been primed subliminally. However, because in everyday life we are often exposed to stimuli of which we are not aware, we believe it is important to assess whether regulatory focus and, more importantly, regulatory fit can be affected by this type of subliminal procedure.

We included both explicit and implicit measures of post-message attitudes because, given the high resistance to change and the strong pressure to conform with an anti-nuclear position characterizing the specific domain investigated here, expected effects might emerge only on implicit rather than explicit, self-reported attitudes. 

On the basis of regulatory fit theory [25,26], we anticipated that when a prevention focus is subliminally primed a message with an avoidance frame, in comparison to a message with an approach frame, will induce a more positive attitude toward nuclear power. On the contrary, we anticipated that when a promotion focus is subliminally primed, a message with an approach frame in comparison to a message with an avoidance frame will induce a more positive attitude toward nuclear power.

### Method

#### Participants

74 students, enrolled at “Sapienza” University of Rome, participated in the study on a voluntary basis. The study was approved by the Ethics Committee of Psychology Research of Sapienza University (n°138-CED01). Participants provided oral informed consent after reading a form. We did not ask for written consent as we wanted to guarantee the anonymity of our participants who were also our students. The consent form was introduced by one of the experimenters who checked that each participant had read and properly understood its content. The ethic committee approved this consent procedure.

#### Focus manipulation

Regulatory focus (either promotion or prevention) was subliminally primed [35] using a foveal presentation of 15 milliseconds of the words *desire, promotion, achievement*, and *gain* to induce a promotion focus, and *prevention, obligation, responsibilities*, and *duties* to induce a prevention focus. Each participant that took part in the experiment was presented with a study on “automatic recognition of word meaning”. Participants were requested to decide whether a noun on the screen had a bad or good meaning. There were 10 positive nouns (*honor, luckiness, diamond, loyalty, freedom, rainbow, honesty, love, peace, paradise*) and 10 negative nouns (*evil, cancer, disease, disaster, poverty, vomit, bomb, rotten, abuse, death*). Actually, the good vs. bad words masked the primed words (promotion vs. prevention) in a total of five blocks of 40 trials each. The first block was intended to train participants to understand which were positive and which were negative nouns. 

After completing the whole experiment, in the debriefing section participants were requested to recall the words they had seen during the “automatic recognition of word meaning” task and to tell whether they remembered words that were difficult to read or recognize. None of the participants named any of the words subliminally presented, or mentioned difficult-to-read words, and therefore we concluded that none of them was aware of the subliminal manipulation.

#### Message framing

The message was presented as a political candidate’s reply to a journalist’s question concerning meeting the future energy needs of the country. This reply had either a strategic approach frame (promotion fit) or a strategic avoidance frame (prevention fit) [strategic avoidance frame shown between square brackets].

*Replacing oil-fired power plants with nuclear ones, that do not produce carbon dioxide and oxides of nitrogen and sulfur – responsible for the ozone depletion and greenhouse effect* – *allows us to increase* [reduce] *the percentage of energy produced* without [with] *harmful consequences for the environment.*

*The production of energy from nuclear power increases the independence* [reduces the dependence] *of the national economy on oil. The coverage of domestic energy needs through nuclear power increases the protection against* [reduces the possibility of] *external shocks on the economy and allows governments to improve the balance* [reduce the imbalance] *of payments with foreign countries. All this translates into the promotion of greater stability* [defense against the instability] *of the national economy. The use of nuclear power increases the independence* [reduces the dependence] *of Western countries on oil produced in Middle Eastern countries characterized by high political instability*.

### Measures

####  Manipulation check

After the subliminal priming task, participants were given a word-completion task consisting of 18 word fragments that could be completed as words related to prevention vs. promotion focus. Then, we counted the number of completed words that were related to prevention or promotion. In the prevention condition, we expected more completed fragments pertaining prevention rather than promotion, whereas in the promotion condition the opposite pattern was expected.

#### Explicit Attitude toward nuclear energy

As an explicit measure of the attitude toward the production of energy through nuclear plants, a semantic differential with three pairs of adjectives (useful-useless, economically advantageous-economically disadvantageous, safe-risky), anchored to a 7 point scale from 1=positive adjective to 7= negative adjective) was used. The first two items were averaged in order to get an index of perceived economic disutility (Cronbach’s α = .87). The third pair of adjectives was used as measure of perceived risk of nuclear plants.

#### Implicit Measure of Attitude toward nuclear energy

We used a single target implicit association test with pictures (ST-IAT) [36,37], to indirectly measure how strongly participants associated nuclear energy pictures with positive and negative words. Participants were asked to classify these pictures and positive and negative words (e.g., pleasure, marvelous, terrible, terrific) with two response keys (left and right) in a congruent and an incongruent block. The congruent block consisted of classifying six nuclear energy pictures and six negative words with the left key, and six positive words with the right key. The incongruent block consisted of classifying six nuclear energy pictures and six positive words with the right key, and six negative words with the left key. Within blocks, all stimuli were presented in random order.

Better performance in terms of shorter response latencies on the congruent block than on the incongruent block was assumed to indicate stronger negative than positive associations with nuclear energy [38]. Each incongruent and congruent block consisted of 40 trials and the practice block consisted of 20 trials. In practice, we used the script developed by Wigboldus et al. available on Inquisit Website (Version 3) [39]. Participants’ D index was computed directly through the website. The higher the D value the more positive the attitude toward nuclear energy.

## Results

### Subliminal priming manipulation check

Results of a 2 (Focus: prevention vs. promotion) by 2 (Type of completed word: related to prevention vs. related to promotion) ANOVA, with the last factor as a repeated measure, showed only a significant Focus by Type of completed word interaction, *F* (1, 72) = 29.704, p <.001. Participants completed more fragments using words related to prevention (*M* = 6.21, *SD* = 1.49) rather than promotion (*M* = 5.34, *SD* = 1.60) after the prevention priming. Conversely, under promotion priming, more fragments were completed with words related to promotion (*M* = 6.42, *SD* = 1.59) rather than prevention (*M* = 5.17, *SD* = 1.60). Our subliminal priming procedure was thus effective.

A 2 (Focus: promotion vs. prevention) by 2 (Frame: approach vs. avoidance) ANOVA was conducted on the implicit measure of attitude toward nuclear energy, the explicit measures of perceived risk and the perceived economic disutility of nuclear energy.

**Figure 1:**
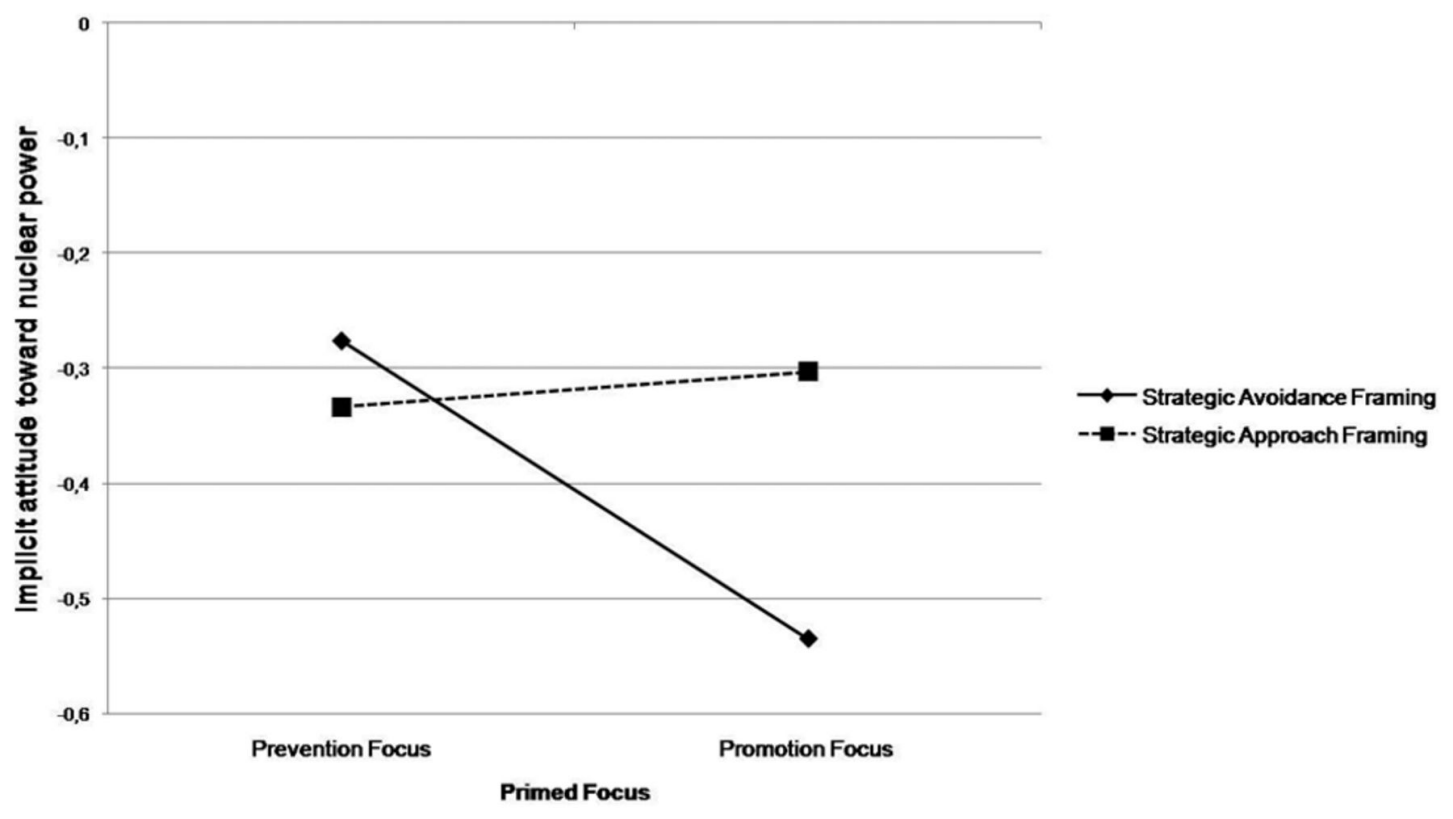
Means of implicit attitudes toward nuclear energy as a function of message framing and regulatory focus.

As to *implicit attitude toward nuclear energy*, there was a significant effect of Focus, *F* (1, 70) = 4.89, *p* <. 05, *η* ^2^ = .06), with more negative attitudes in promotion (*M* = - .419, *SD* = .27) than in prevention condition (*M* = -.305, *SD* = .19). Also, a nearly significant effect of Frame emerged, *F* (1, 70) = 2.86; *p* < .09, *η* ^2^ = .05 with more negative attitudes in the strategic avoidance framing (*M* = -.402, *SD* = .25), than in the strategic approach framing (*M* = -.319, *SD* = .22). More central to our purpose, the expected Focus by Frame interaction was significant *F* (1, 70) = 7.91; *p* < .01, *η* ^2^ = .10), As shown in Figure 1, as expected, in the prevention focus condition, more negative attitude toward nuclear energy emerged in the strategic approach framing (*M* = -.334*, SD* = .14) than in the strategic avoidance framing (*M* = -.276, *SD* = .23). However, simple effects analysis showed that this difference was not significant (*F <1*). As predicted, the opposite pattern emerged for the promotion focus condition. More specifically, participants in the strategic approach framing had a less negative attitude (*M* = -.303, *SD* = .25) than those in the strategic avoidance framing (*M* = -.532, *SD* = .21), with this difference being significant, *F* (1, 70) = 9.88, *p* < .002. 

Furthermore, according to simple effects analysis, the message with strategic avoidance frame was significantly more effective among participants in the prevention focus condition than for those in the promotion focus condition, F (1, 70) = 12.62, *p* < .001, while no difference emerged for the message with strategic approach frame (*F* < 1). 

*Perceived risk* of nuclear energy was not affected by the Focus by Frame interaction, or by main effects of either of the two variables (all *F*s ≤ 1). In general, participants expressed an elevated perception of risk deriving from nuclear energy (M = 5.2, *SD* = 1.85).

*Perceived economic disutility* of nuclear energy was not affected by the Focus by Frame interaction (*F* < 1), nor by main effects of either of the two variables (for focus, *F* < 1, and for framing *F* (1, 70) = 1.60, *p =* ns. The general mean of nuclear energy economic disutility was 4.4 (SD = 1.28), which means that nuclear energy was perceived as being slightly more useless than useful.

### Discussion

The results of this study support the positive persuasive impact of regulatory fit induced by subliminal priming of regulatory focus combined with framing of the message, even for a very controversial political issue such as nuclear power. Our findings, however, are limited to negative implicit attitude toward nuclear energy and are stronger among participants in the promotion focus condition. The lack of effects at level of explicit perceived risk can be explained by the very content of the message that was focused on economic utility of nuclear power production, and did not provide any information concerning risk reduction, whereas the lack of effect on explicit judgment of economic utility may be due to the already mentioned difficulty to increase nuclear power support [34]. Therefore, in Study 2 we focused solely on explicit measures regarding a social issue (i.e., immigration) which is typically less resistant to persuasion than nuclear energy. 

Another limit of this study is that it did not explore whether regulatory fit affects not only attitudes toward a specific political issue but also toward the message source that is the political candidate, which, in the political domain, is often the main goal of the communication. This was the focus of Study 2.

## Study 2

In study 2, we aimed at assessing the impact of regulatory fit on recipients’ attitude both on recipients’ attitude toward a political issue and on the evaluation of the political candidate who is the source of the message. In order to test the impact of regulatory fit on persuasive efficacy of messages across different political issues, we chose to use a message concerning immigration policies.

Just like the other countries in Southern Europe, Italy has, in the last two decades, rapidly and unexpectedly changed from a country of emigration into one of immigration. In just 15 years as a country of immigration, Italy now occupies the fourth position when it comes to the number of immigrants hosted by European countries.

Rising immigrant population, combined with adverse economic trends, the presence of right-wing populism, and the preoccupation with Muslim terrorism, has placed immigration and asylum on the political agenda over the course of the last few years. Immigrants are commonly portrayed in the media, public discourse, and private debate as competing for employment and housing, unfairly or illegally drawing on public welfare resources, and being associated with criminality.

The public opinion, which initially was an attitude of ‘social tolerance’ towards immigrants, has become hostile and xenophobic in recent years [40,41]. It is also not uncommon that immigrants, and in particular those with irregular residence permits, are portrayed in the mass media and in political speeches as criminals and as being involved in a number of clandestine activities. Therefore, the issue of immigration policies has been frequently at the center of the political debate and candidates have frequently expressed their opinions and suggested the best strategies to cope with the problem. In this study, we used an ad hoc constructed message, presented as a candidate’s answer to an interview, that explicitly suggested accepting only 20% of people who intended to come into Italy and framed this content either in a positive (accept 20%) or in a negative (refuse 80%) frame.

On the basis of regulatory fit theory [25,26], we anticipated that when a prevention focus is subliminally primed a message with a negative frame (refuse 80%), in comparison to a message with an approach frame (accept 20%), will induce a more positive attitude toward the message content and the political candidate. On the contrary, we anticipated that when a promotion focus is subliminally primed a message with a positive frame, in comparison to a message with a negative frame, will induce a more positive attitude toward the message content and the political candidate. Furthermore, we anticipated that the positive impact of regulatory fit on the evaluation of the political candidate and on the intention to vote for him/her would be mediated by the positive reactions to the message.

### Method

#### Participants

60 undergraduate students, enrolled at “Sapienza” University of Rome, participated in the study on a voluntary basis. The study was approved by the Ethics Committee of Psychology Research of Sapienza University (n° 139-CED01). Participants provided oral informed consent after reading a form. We did not ask for written consent as we wanted to guarantee the anonymity of our participants who were also our students. The consent form was introduced by one of the experimenters who checked that each participant had read and properly understood its content. The ethics committee approved this consent procedure.

#### Focus manipulation

Regulatory focus was subliminally primed as in Study 1.

#### Message framing

The message was presented as in Study 1 and had either a strategic approach frame (promotion fit) or a strategic avoidance frame (prevention fit) [strategic avoidance frame shown between square brackets].

*I think it is necessary to ensure* [to avoid not having] *an adequate number of immigrants to foster a positive trend* [to avoid the development of a negative trend] *of the national economy. As a percentage on the current number of people requesting to come into Italy, this will result in accepting 20%* [in rejecting only 80%] *of such requests. To do so, I will promote* [make sure not to hinder] *the conclusion of bilateral agreements with other countries in order to achieve programmed arrivals* [to avoid unplanned arrivals] *of people* with [without] *professional qualifications suitable to the needs of our companies. Moreover, I will endeavor to promote the integration of second generation immigrants into the educational system* [to avoid the production of barriers to the education of second generation immigrants] *and to facilitate their full integration* [to counter the risk of a lack of their integration] *into the civic and political life of our nation.*

#### Measures

The impact of the political messages was assessed measuring both evaluations of the message content and evaluations of the candidate. As far as the first were concerned, participants were requested to judge: perceived validity of message arguments (on a 10 point scale), and estimated likelihood of positive consequences of the election of the candidate for the immigration issue (on a seven point scale). Evaluation of the candidate was measured in terms of: perceived energy and trustworthiness, positive global evaluation (on a 10 point scale), and intention to vote (on a 4 point scale). An “energy” rating (α = .68) was obtained by asking (on seven point scales) to what extent the candidate was decisive, energetic, dynamic, and tenacious. An “honesty” rating (α = .80) was obtained by asking (on seven point scales) to what extent the candidate was honest, loyal, sincere, and trustworthy.

## Results

Data were analyzed performing several 2 (Focus: promotion vs. prevention) by 2 (Frame: approach vs. avoidance) Anovas. 

*Perceived validity of message arguments* was affected only by Frame, *F* (1, 56) = 11.10, *p* < .002, *η* ^2^ = .16): participants exposed to the strategic approach-framed message perceived them to be stronger (*M* = 3.87, *SD* = .97) than participants exposed to the strategic avoidance-framed message (*M* = 2.83, *SD* = 1.39).

**Figure 2:**
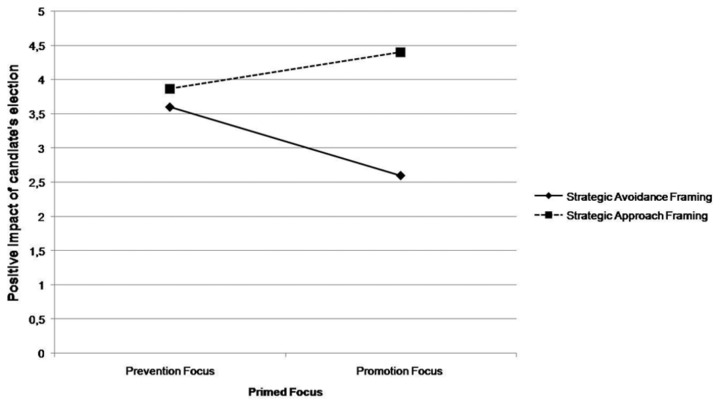
Means of prevision of a positive impact of the election of the candidate on the immigration as a function of message framing and regulatory focus.

As to *anticipation of a positive impact of the election of the candidate for the immigration*, the main effect of Frame, *F* (1, 56) = 8.58, *p* < .01, *η* ^2^ = .13, was qualified by the predicted two-way interaction, *F* (1, 56) = 4.72, *p* < .05, *η* ^2^ = .08). As shown in Figure 2, and confirmed by post-hoc comparisons, in the promotion focus condition participants anticipated more positive impact of the candidate on the immigration under approach (*M* = 4.40, *SD* = 1.40) rather than avoidance framing (*M* = 2.6, *SD* = 1.18), *F* (1, 56) = 13.08, *p* <.002. 

 Under prevention focus means of approach and avoidance frame were not significantly different, *F* < 1. Consistent with the fit explanation, simple effect analysis showed that the strategic avoidance frame was significantly more effective, *F* (1, 56) = 4.02, *p* <.05, among participants in the prevention focus condition (*M* = 3.6, *SD* = 1.55) than for those in the promotion focus condition (*M* = 2.6, *SD* = 1.18). Despite means being in the predicted direction, when the approach frame was adopted no significant differences emerged between promotion and prevention focus condition, *F* (1, 56) = 1.14, *p* = ns. 

*Energy* attributed to the candidate did not vary as a function of Frame, Focus or their interaction. *Honesty* attributed to the candidate was affected only by Frame, *F* (1, 56) = 18.49, *p* <.001, *η* ^2^ = .25: the source of the strategic approach-framed message was judged as more honest (*M* = 4.17, *SD* = .82) than the source of the strategic avoidance-framed message (*M* = 3.14, *SD* = 1.02). 

**Figure 3:**
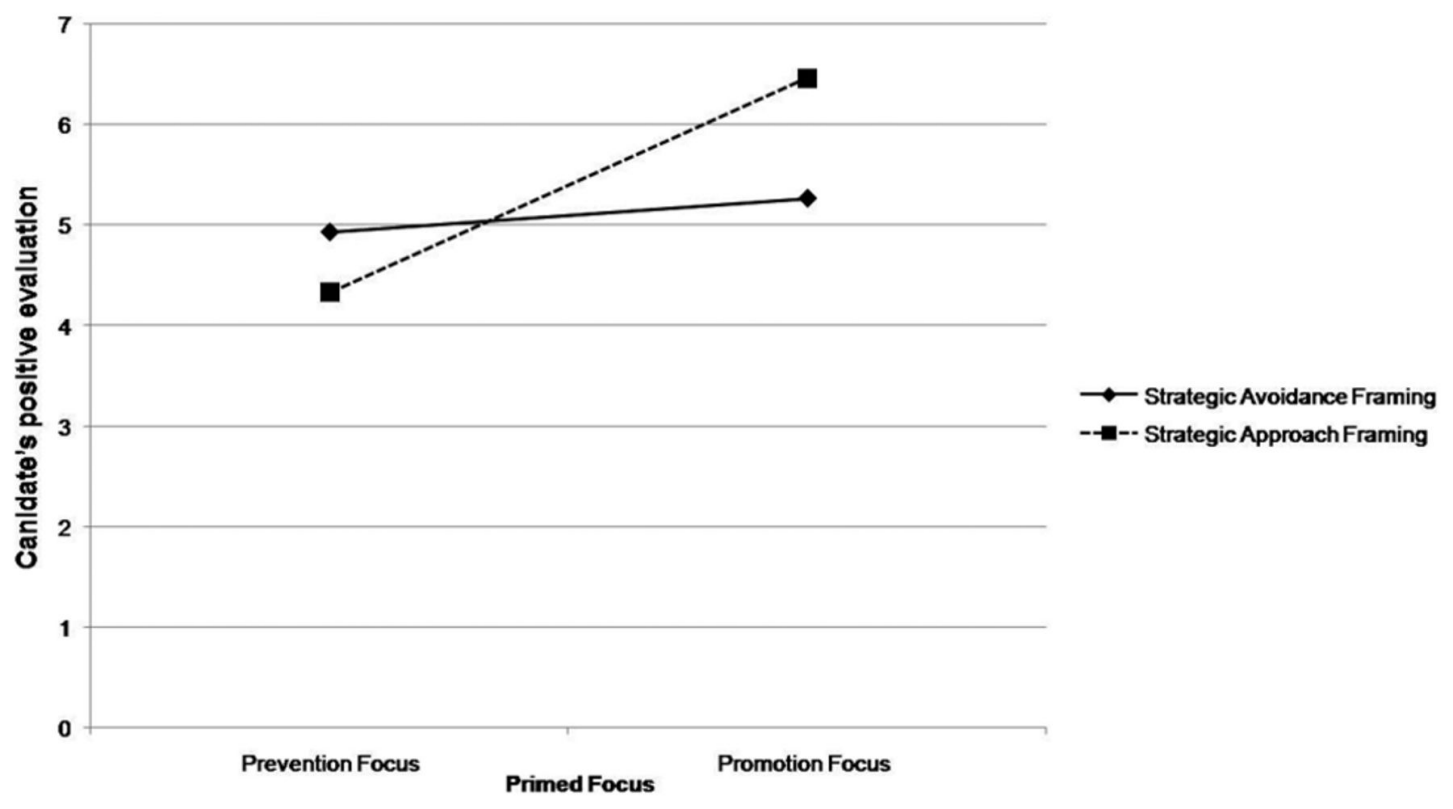
Means of positive global evaluation of the political candidate as a function of message framing and regulatory focus.

For the *positive global evaluation of the political candidate*, there was both a main effect of Frame *F* (1, 56) = 8.25, *p* <. 01, *η* ^2^ = .13; and the hypothesized Focus by Frame interaction *F* (1, 56) = 4.39, *p* <. 05, *η* ^2^ = .07. As can be seen in Figure 3, in the promotion condition, global evaluation of the political candidate was significantly more positive for participant under approach frame (*M* = 6.47, *SD* = 1.19) rather than avoidance frame (*M* = 4.33, *SD* = 1.15), *F* (1, 56) = 12.34, *p* <. 001. Again, means in the prevention condition, although consistent with predictions, were not significantly different, *F* (1, 56) = .98, *p* = ns. As expected, the message with strategic approach frame was significantly more effective in promoting a positive global evaluation of the candidate among participants in the promotion focus condition (*M* = 6.47, *SD* = 1.19) than for those in the prevention focus condition (*M* = 4.93, *SD* = 2.22), *F* (1, 56) = 3.90, *p* <. 05. Under avoidance framing, an increase in positive evaluation among participants in the prevention (vs. promotion) focus emerged, but the difference was not significant, *F* (1, 56) = .98, *p* = ns.

**Figure 4:**
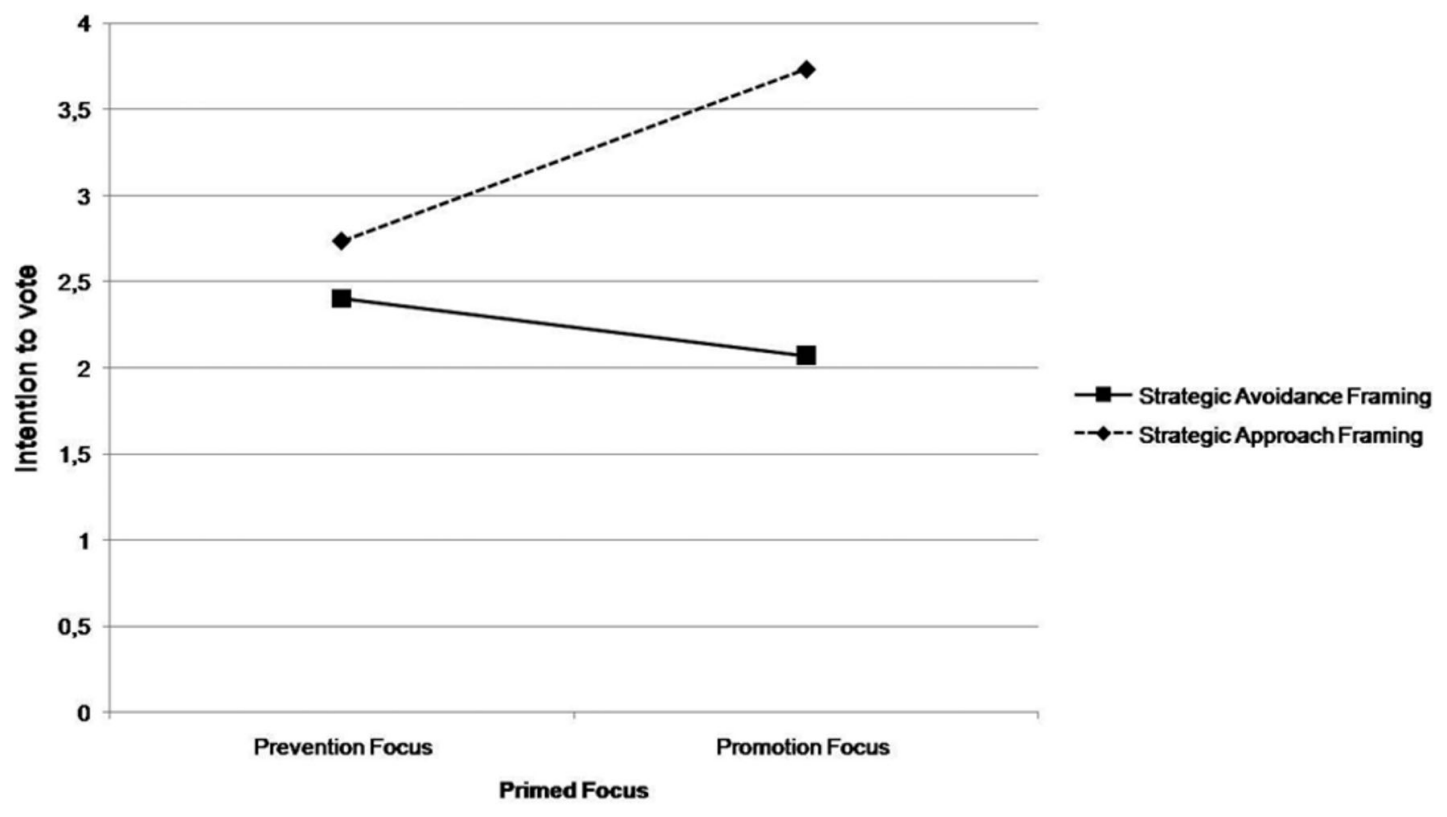
Means of intention to vote for the political candidate as a function of message framing and regulatory focus.

A similar pattern emerged with respect to *intention to vote for the political candidate*. More specifically, the ANOVA revealed a main effect of Frame, *F* (1, 56) = 9.77, *p* <. 01. *η* ^2^ = .15, qualified by the interaction, *F* (1, 56) = 4.32, *p* <. 05, *η* ^2^ = .07. As can be noted in Figure 4, in the promotion condition the intention to vote for the political candidate was stronger for those presented with the approach (*M* = 3.73, *SD* = .79) rather than avoidance (*M* = 2.07, *SD* = 1.22) frame with this difference being significant, *F* (1, 56) = 13.50, *p* <. 001. No difference emerged in the prevention focus condition, *F* < 1. 

Again, consistent with the expected fit pattern, under approach framing the message was more effective, *F* (1, 56) = 4.86, *p* <. 05, among participants in the promotion focus condition (*M* = 3.73, *SD* = .79) than among those in the prevention focus condition (*M* = 2.73, *SD* = 1.33), while the message with strategic avoidance frame was not significantly more effective for participants in the prevention focus condition than for those in the promotion focus condition, F (1, 56) = .540, p = .47.

Given that likelihood of positive consequences of the election of the candidate was significantly correlated both with positive global evaluation of the candidate (*r* = .77, *p* < .01) and with the intention to vote for him/her (*r* = .63, *p* < .01), we performed two bootstrap moderated mediation analyses [42], in order to assess whether this measure of attitude toward the issue mediated the impact of fit on candidate’s reputation (i.e., global evaluation and intention to vote). 

In the first moderated mediation analysis, having positive global evaluation of the candidate as a dependent variable, the 95% bias-correct confidence interval for indirect effect of the fit effect did not include zero (Lower = .0380, Upper = .7046), indicating that the likelihood of positive consequences of the election of the candidate for immigration mediated the impact of the interaction between Focus and Frame on positive global evaluation of the political candidate.

In the second moderated mediation analysis, having intention to vote as the dependent variable, the 95% bias-correct confidence interval for indirect effect of the interaction did not include zero (Lower = .1062, Upper = 1.7737), indicating that the likelihood of positive consequmences of the election of the candidate for immigration was responsible for the impact of the interaction between Focus and Frame on intention to vote for the candidate.

## General Discussion

Previous studies have shown that persuasive messages (both commercial and otherwise) are particularly effective if the content of a message suits the recipients’ regulatory focus [15-16-17-19- 20-29-30]. Results of the present studies show that this is also true for messages concerning different issues of political relevance, such as usage of nuclear power energy and immigration policies. Implicit attitudes toward nuclear energy, among participants belonging to a population characterized by a strongly negative attitude toward this issue, became significantly more positive after exposure to a message describing the economic benefits of nuclear power when the frame of this message fits recipients’ regulatory focus. Interestingly, this effect did not extend to explicit attitudes, which demonstrates the importance of measuring some attitudes implicitly, especially for politically controversial topic. 

The results of Study 2 show that regulatory fit can also impact attitudes and behavioral intentions regarding a hypothetical candidate that is presented as the message source. In regulatory fit conditions, participants anticipated more positive outcomes concerning immigration from the candidate’s election, evaluated the candidate more positively, and had a stronger intention to vote for the candidate. Importantly, these persuasion effects of regulatory fit occurred when promotion and prevention were experimentally induced by subliminal priming. To our knowledge, this is the first demonstration that fit can be created in this way and have persuasion effects. Furthermore, in Study 2, moderated mediation analyses show that regulatory fit impact on evaluation of the candidate and on intention to vote was mediated by expected outcomes concerning the specific issue. In other words, regulatory fit increases positive expectations concerning the amelioration of immigration problems by the action of the candidate and, as a consequence, the positive evaluation of the candidate and the intention to vote for him/her. Present work also points out how framing of political persuasive messages can be moderated by regulatory focus. This has obvious practical implications. For example, according to the dominant regulatory focus of the audience, a politician can change the frame of the message. Alternatively, if the dominant regulatory focus is unknown, as in most cases, a regulatory focus can be subliminally or supraliminally induced and subsequently a frame that fits the induced focus can be adopted. Our research thus provides a powerful conceptual tool for political persuasion. 

As compared to previous researches on regulatory fit [25,26] our results do not show the typical preference reversal between promotion and prevention focus condition. In our case, fit effect is stronger under promotion rather than prevention condition. This finding might be a consequence of the fact that, for the first time, fit originated from subliminally induced regulatory focus. Although this procedure was effective, as manipulation check confirmed, a fit based on subliminal induction of regulatory focus might well have different properties that are still largely unknown as compared to more traditional types of fit. For example, we know that fit effects are based on a ‘feeling right’ experience [15]. That is, in the persuasion domain, individuals experiencing fit feel right about the process of being persuaded and misattribute the positive affective experience to the message itself or to the source. This affective misattribution might be less intense under prevention focus. Research indeed showed that a prevention focus is accompanied by an analytical, concrete, and detailed processing style [43,44] which might prevent biased misattributions underlying regulatory fit. Future research should explore the intriguing hypothesis that misattribution of feeling right due to fit might be less intense when the prevention focus is subliminally induced. 

At a more theoretical level, regulatory fit effects emerged here can also be interpreted under a goal-systematic perspective. Kruglanski, indeed, suggested that fit can be seen as “a match between a person's activity and his/her (background) “process goal” to pursue a (focal) attainment goal in a desired manner” (p. 11) [45] that is by using either eager or vigilant strategies. However, Avnet and Higgins clarified that regulatory fit theory differs from other lines of research about goal activation [46], as it “is not concerned with the relationship between a goal and the means to that goal. Instead it is concerned with the relationship between a person's current goal orientation and weather the means to goal pursuit sustains or disrupts that orientation” [47]. The focus is thus more on the way a person pursues a goal rather than on the specific goal contents. This is an important distinction because it remarks how fit is a general mechanism that is not restricted to regulatory focus. Recent research for example showed that fit might be present when locomotion and assessments – two regulatory orientation pertaining how goals should be achieved – are matched with pertinent means to achieve a goal [48,49]. 

In conclusion, the results of these studies show that regulatory fit can benefit political communication both in terms of attitude change, at implicit and explicit level, and in terms of enhancing the candidate’s reputation and the likelihood of voting for the candidate. The effects obtained in these studies are of particular interest because recipients’ regulatory focus was not measured as a chronic personality characteristic, but was subliminally primed before delivering the message. As experimental research on regulatory focus has used several other procedures to situationally induce either a promotion or a prevention focus, the present results demonstrate that fit effects on persuasion are not restricted to such procedures. This is noteworthy and opens new questions for future research especially pertaining differences and similarities between fit obtained subliminally and other types of fit. 

## References

[1] Katz E (1973) Platforms and windows: Broadcasting’s role in election campaigns.Journal: Q 48: 304-314

[2] Krosnick JA, Brannon LA (1993) The impact of the gulf war on the ingredients of presidential evaluations: Multidimensional effects of political involvement. Am Polit Sci Rev 87: 963-975. doi:10.2307/2938828.

[3] Lockerbie B (1991) The temporal pattern of economic evaluations and vote choice in senate elections. Public Choice 69: 279-294. doi:10.1007/BF00123865.

[4] Bizer GY, Petty RE (2005) How we conceptualize our attitudes matters: The effects of valence framing on the resistance of political attitudes. Polit Psychol 26: 553-568. doi:10.1111/j.1467-9221.2005.00431.x.

[5] Jost JT, Sidanius J (2004) Political Psychology: Key Readings. New York: Psychology Press. 497 pp.

[6] Pyszczynski TA, Solomon S, Greenberg J (2003) In the wake of 9/11: The psychology of terror. Washington, DC: American Psychological Association. 227 pp.

[7] Price V, Tewksbury D, Powers E (1997) Switching trains of thought: The impact of the news frames on readers’ cognitive responses. Commun Res 24: 481-506. doi:10.1177/009365097024005002.

[8] Reese S, Gandy O, Grant A (2001) Framing public life. (Eds.) Mahwah, NJ: Erlbaum . 396 p

[9] Weaver DH (2007) Thoughts on agenda setting, framing, and priming. J Commun 57: 142-147. doi:10.1111/j.1460-2466.2006.00333.x.

[10] Tversky A, Kahneman D (1986) Rational choice and the framing of decisions. J Bus, 59: 251- 278. doi:10.1086/296365.

[11] Shen F (2004) Chronic accessibility and individual cognitions: Examining the effects of message frames in political advertisements. J Commun 54: 123-127. doi:10.1111/j.1460-2466.2004.tb02617.x.

[12] Druckman J (2001) Using credible advice to overcome framing effects. J Law Econ Organ 17: 62-82. doi:10.1093/jleo/17.1.62.

[13] Higgins ET (1997) Beyond pleasure and pain. Am Psychol 52: 1280-1300. doi:10.1037/0003-066X.52.12.1280. PubMed: 9414606. 9414606

[14] Higgins ET (1998) Promotion and prevention: Regulatory focus as a motivational principle. In: Zanna MP Advances in experimental social psychology. New York: Academic Press pp. 1-46.

[15] Cesario J, Grant H, Higgins ET (2004) Regulatory fit and persuasion: Transfer from “feeling right”. J Pers Soc Psychol 86: 388-404. doi:10.1037/0022-3514.86.3.388. PubMed: 15008644. 15008644

[16] Evans LM, Petty RE (2003) Self-guide framing and persuasion: Responsibly increasing message processing to ideal levels. Pers Soc Psychol Bull 29: 313-324. doi:10.1177/0146167202250090. PubMed: 15273009. 15273009

[17] Florack A, Scarabis M (2006) How advertising claims affect brand preferences and category-brand associations: The role of regulatory fit. Psychol Mark 23: 741-755. doi:10.1002/mar.20127.

[18] Lee AY, Aaker JL (2004) Bringing the frame into focus: The influence of regulatory fit on processing fluency and persuasion. J Pers Soc Psychol 86: 205-218. doi:10.1037/0022-3514.86.2.205. PubMed: 14769079. 14769079

[19] Pham MT, Avnet T (2004) Ideals and thoughts and the reliance on affect versus substance in persuasion. J Consum Res 30: 503-518. doi:10.1086/380285.

[20] Spiegel S, Grant-Pillow H, Higgins ET (2004) How regulatory fit enhances motivational strength during goal pursuit. Eur J Soc Psychol 34: 39-54. doi:10.1002/ejsp.180.

[21] Higgins ET, Spiegel S (2004) Promotion and prevention strategies for self-regulation: A motivated cognition perspective. In: Baumeister RF Vohs KD Handbook of self-regulation: Research, theory and applications. New York: Guilford Press pp. 171-187.

[22] Lee AY, Aaker JL, Gardner WL (2000) The pleasures and pains of distinct self-construals: The role of interdependence in regulatory focus. J Pers Soc Psychol 78: 1122-1134. doi:10.1037/0022-3514.78.6.1122. PubMed: 10870913. 10870913

[23] Freitas AL, Liberman N, Higgins ET (2002) Regulatory fit and resisting temptation during goal pursuit. J Exp Soc Psychol 38: 291-298. doi:10.1006/jesp.2001.1504.

[24] Liberman N, Idson LC, Camacho CJ, Higgins ET (1999) Promotion and prevention choices between stability and change. J Pers Soc Psychol 77: 1135-1145. doi:10.1037/0022-3514.77.6.1135. PubMed: 10626368. 10626368

[25] Higgins ET (2000) Making a good decision: Value from fit. Am Psychol 55: 1217-1230. doi:10.1037/0003-066X.55.11.1217. PubMed: 11280936. 11280936

[26] Higgins ET (2002) How self-regulation creates distinct values: The case of promotion and prevention decision making. J Consum Psychol 12: 177-191. doi:10.1207/S15327663JCP1203_01.

[27] Aaker JL, Lee AY (2001) “I” seek pleasures and “we” avoid pains: The role of self- regulatory goals in information processing and persuasion. J Consum Res 28: 33-49. doi:10.1086/321946.

[28] Camacho CJ, Higgins ET, Luger L (2003) Moral value transfer from regulatory fit: What feels right is right and what feels wrong is wrong. J Pers Soc Psychol 84: 498-510. doi:10.1037/0022-3514.84.3.498. PubMed: 12635912. 12635912

[29] Holler M, Hoelzl E, Kirchler E, Leder S, Mannetti L (2008) Framing of information on the use of public finances, regulatory fit of recipients and tax compliance. J Econ Psychol 29: 597-611. doi:10.1016/j.joep.2008.01.001. PubMed: 20495689. 20495689 PMC2874666

[30] Leder S, Mannetti L, Hölzl E, Kirchler E (2010) Regulatory fit effects on perceived fiscal exchange and tax compliance. J Socio Econ 39: 271-277. doi:10.1080/03085141003620170. PubMed: 20890461. 20890461 PMC2948557

[31] Boldero JM, Higgins ET (2011) Regulatory Focus and Political Decision Making: When People Favor Reform Over the Status Quo. Polit Psychol 32: 399-418. doi:10.1111/j.1467-9221.2010.00814.x.

[32] Janoff-Bulman R (2009) To provide or protect: Motivational bases of political liberalism and conservatism. Psychol Inq 20: 120-128. doi:10.1080/10478400903028581.

[33] Scholer AA, Higgins ET (2008) Distinguishing levels of approach and avoidance: An analysis using regulatory focus theory. In: Elliot AJ Handbook of approach and avoidance motivation. New York: Psychology Press pp. 489-503.

[34] Whitfield SRE, Dan A, Dietz T (2009) The future of nuclear power: Value orientations and risk perception. Risk Anal 29: 425-443. doi:10.1111/j.1539-6924.2008.01155.x. PubMed: 19000075. 19000075

[35] Bargh JA, Chartrand TL (2000) The mind in the middle: A practical guide to priming and automaticity research. In: Reis H Judd CM Handbook of research methods in social psychology. New York: Cambridge University Press pp. 253-285.

[36] De Liver Y, Van der Pligt J, Wigboldus D (2007) Positive and negative associations underlying ambivalent attitudes. J Exp Soc Psychol 43: 319-326. doi:10.1016/j.jesp.2006.02.012.

[37] Bluemke M, Friese M (2008) Reliability and validity of the Single-Target IAT (ST-IAT): Assessing automatic affect towards multiple attitude object. Eur J Soc Psychol 38: 977-997. doi:10.1002/ejsp.487.

[38] Greenwald AG, McGhee DE, Schwartz JKL (1998) Measuring individual differences in implicit cognition: The Implicit Association Test. J Pers Soc Psychol 74: 1464–1480. doi:10.1037/0022-3514.74.6.1464. PubMed: 9654756. 9654756

[39] Wigboldus DHJ, Holland RW, van Knippenberg A (2006) Single Target Implicit Associations, (unpublished manuscript)

[40] Sniderman MP, Peri P, De Figueiredo JPR Jr, Piazza T (2000) The Outsider Prejudice and Politics in Italy. Princeton: Princeton University Press. 219 pp.

[41] Triandafyllidou A (1999) Nation and immigration: A study of the Italian press discourse. Soc Identities 5: 65-88. doi:10.1080/13504639951626.

[42] Hayes AF (2012) PROCESS: A versatile computational tool for observed variable mediation, moderation, and conditional process modeling [White paper]. Available: http://www.afhayes.com/public/process2012.pdf. Accessed 12 June 2013.

[43] Förster J, Higgins ET (2005) How Global Versus Local Perception Fits Regulatory Focus. Psychol Sci 16: 631-636. doi:10.1111/j.1467-9280.2005.01586.x. PubMed: 16102066. 16102066

[44] Roskes M, Elliot AJ, Nijstad BA, De Dreu CKW (2013) Avoidance Motivation and Conservation of Energy. Emot Rev 5: 308-311. doi:10.1177/1754073913477517.

[45] Kruglanski AW (2006) The nature of fit and the origins of 'feeling right': A goal-systemic perspective. J Mark Res 43: 11-14. doi:10.1509/jmkr.43.1.11.

[46] Kruglanski AW, Shah JY, Fishbach A, Friedman R, Chun WY, Sleeth-Keppler D (2002) A theory of goal systems. In: Zanna MP Advances in experimental social psychology. San Diego: Academic Press pp. 331- 378.

[47] Avnet T, Higgins ET (2006) How Regulatory Fit Affects Value in Consumer Choice and Opinions. J Mark Res 4: 31-10.

[48] Avnet T, Higgins ET (2003) Locomotion, assessment, and regulatory fit: Value transfer from 'how' to 'what'. J Exp Soc Psychol 39: 525-530. doi:10.1016/S0022-1031(03)00027-1.

[49] Mannetti L, Giacomantonio M, Higgins ET, Pierro A, Kruglanski AW (2010) Tailoring visual images to fit: Value creation in persuasive messages. Eur J Soc Psychol 40: 206-215.

